# Frequency and predictors of complication clustering within 30 days of spinal fusion surgery: a study of children with neuromuscular scoliosis

**DOI:** 10.1007/s43390-023-00813-8

**Published:** 2024-02-09

**Authors:** Sujay Rajkumar, Rajiv R. Iyer, Lauren Stone, Michael P. Kelly, Jillian Plonsker, Michael Brandel, David D. Gonda, Marcus D. Mazur, Daniel S. Ikeda, Donald J. Lucas, Pamela M. Choi, Vijay M. Ravindra

**Affiliations:** 1 Drexel University College of Medicine, Philadelphia, PA USA; 2 Department of Neurosurgery, Clinical Neurosciences Center, University of Utah, 175 N. Medical Drive East, Salt Lake City, UT 84132 USA; 3 Division of Pediatric Neurosurgery, Primary Children’s Hospital, Salt Lake City, UT USA; 4 Department of Neurosurgery, University of California San Diego, San Diego, CA USA; 5 Department of Orthopedics, Rady Children’s Hospital and University of California-San Diego Medical Center, San Diego, CA USA; 6 Division of Pediatric Neurosurgery, Rady Children’s Hospital, San Diego, CA USA; 7 Department of Neurosurgery, Walter Reed National Military Medical Center, Bethesda, MD USA; 8 Division of Pediatric Surgery, Department of General Surgery, Naval Medical Center San Diego, San Diego, CA USA; 9 Department of Neurosurgery, Naval Medical Center San Diego, San Diego, CA USA

**Keywords:** Pediatric, Spinal deformity, Neuromuscular scoliosis, Fusion, Complication, Co-occurrence, National Surgical Quality Improvement Program

## Abstract

**Purpose:**

There is limited information on the clustering or co-occurrence of complications after spinal fusion surgery for neuromuscular disease in children. We aimed to identify the frequency and predictive factors of co-occurring perioperative complications in these children.

**Methods:**

In this retrospective database cohort study, we identified children (ages 10–18 years) with neuromuscular scoliosis who underwent elective spinal fusion in 2012–2020 from the National Surgical Quality Improvement Program-Pediatric database. The rates of co-occurring complications within 30 days were calculated, and associated factors were identified by logistic regression analysis. Correlation between a number of complications and outcomes was assessed.

**Results:**

Approximately 11% (709/6677 children with neuromuscular scoliosis undergoing spinal fusion had co-occurring complications: 7% experienced two complications and 4% experienced ≥ 3. The most common complication was bleeding/transfusion (80%), which most frequently co-occurred with pneumonia (24%) and reintubation (18%). Surgical time ≥ 400 min (odds ratio (OR) 1.49 [95% confidence interval (CI) 1.25–1.75]), fusion ≥ 13 levels (1.42 [1.13–1.79]), and pelvic fixation (OR 1.21 [1.01, 1.44]) were identified as procedural factors that independently predicted concurrent complications. Clinical risk factors for co-occurring complications included an American Society of Anesthesiologist physical status classification ≥ 3 (1.73 [1.27–2.37]), structural pulmonary/airway abnormalities (1.24 [1.01–1.52]), impaired cognitive status (1.80 [1.41–2.30]), seizure disorder (1.36 [1.12–1.67]), hematologic disorder (1.40 [1.03–1.91], preoperative nutritional support (1.34 [1.08–1.72]), and congenital malformations (1.20 [1.01–1.44]). Preoperative tracheostomy was protective against concurrent complications (0.62 [0.43–0.89]). Significant correlations were found between number of complications and length of stay, non-home discharge, readmissions, and death.

**Conclusion:**

Longer surgical time (≥ 400 min), fusion ≥ 13 levels and pelvic fixation are surgical risk factors independently associated with co-occurring complications, which were associated with poorer patient outcomes. Recognizing identified nonmodifiable risk factors might also be important for preoperative planning and risk stratification of children with neuromuscular scoliosis requiring spinal fusion.

**Level of evidence:**

Level IV evidence.

**Supplementary Information:**

The online version contains supplementary material available at 10.1007/s43390-023-00813-8.

## Introduction

Surgical intervention is commonly used to stabilize pediatric spinal deformities to improve patients’ pain, daily function, and cosmetic appearance [16]. Although specific goals differ by patient needs, the broad aims of spinal corrective surgery are to restore regional and global alignment, prevent curve progression, prevent or halt deterioration of cardiopulmonary function, and establish a solid fusion [1, 17]. Because spinal fusion procedures for deformity are mostly elective, considerations for surgery include a firm understanding of potential complications and factors that may contribute to the morbidity and mortality of a child undergoing these procedures [21]. The literature demonstrates a moderate risk of complications after pediatric spinal corrective surgery; 15% of patients experience non-neurologic issues and 1% experience neurologic sequelae. For children with adolescent idiopathic scoliosis, the rates are 5–23% [7, 19], but children with neuromuscular disease represent a higher-risk population. Therefore, a comprehensive assessment of factors that may adversely affect a patient’s course of recovery (independently or concurrently) after fusion surgery is warranted.

Complications that cluster can lead to increased morbidity and may have a significant impact on hospital length of stay. Bortz et al. [5] demonstrated a 6% rate of concurrent complications in adults undergoing correction for adult spinal deformity, but there have been no studies examining co-occurring or concurrent post-fusion complications among pediatric patients with neuromuscular scoliosis.

To further stratify risk in this group, we assessed whether relationships exist among complications and, specifically, whether complications cluster. Using a large national surgical quality database, we analyzed rates of independent and co-occurring postoperative complications after spinal fusion surgery for pediatric patients with neuromuscular scoliosis. We hypothesized that certain patient- and procedure-related factors predispose children with neuromuscular scoliosis to concurrent complications when undergoing surgery for spinal fusion. Additionally, given increased frailty in the neuromuscular scoliosis population, we also expected to observe a higher incidence of postoperative complications compared with children without neuromuscular disease.

## Materials and methods

### Data source

This retrospective cohort study used the American College of Surgeons National Surgical Quality Improvement Program Pediatric (NSQIP-P) database. NSQIP-P tracks and audits 30-day perioperative and surgical outcomes for individuals ≤ 18 years. Variables were collected based on a systematic sampling process that allows for proportional diversity in selection by using an 8-day sampling cycle from more than 60 pediatric institutions [15]. The NSQIP-P data are audited and validated on a continual basis and organized into participant user files for use in quality improvement and outcomes research. For the current investigation, the 2012–2020 data were used [2]. Because NSQIP-P participant user file datasets do not have patient identifying information, the study was exempt from ethics review as determined by the local institutional review board and human subjects protection office.

### Study design and inclusion criteria

Patients aged 10–18 years of age who underwent spinal fusion with a diagnosis of neuromuscular scoliosis were included; children who underwent growth preservation surgery or fusionless operations were excluded. The Current Procedural Terminology (CPT) codes used for identification included 22800, 22802, and 22804 (arthrodesis, posterior, for spinal deformity, with or without case; up to 6 vertebral segments, 7–12 vertebral segments, 13 or more vertebral segments, respectively) and 22808, 22810, and 22812 (arthrodesis, anterior, for spinal deformity with or without case; 2–3 vertebral segments, 4–7 vertebral segments, 8 or more vertebral segments, respectively). The secondary CPT code 22848 (pelvic fixation—attachment of caudal end of instrumentation to pelvic bony structures) was also included. In the neuromuscular population, pelvic fixation is common and can add morbidity [8, 14, 28, 31], so the CPT code 22848 was queried and used as a secondary code in applicable cases. Otherwise, only primary codes were used for the analysis. Patients who underwent emergent surgery or had prior surgery within 30 days were excluded from the analysis.

The primary outcome was co-occurring complications (≥ 2 complications occurring intraoperatively or perioperatively affecting multiple organ systems) within 30 days. Secondary outcomes included infectious complications, non-home discharge, days of mechanical ventilation, readmission, length of stay, and death. Broadly, complications were defined by NSQIP definitions and recorded events.

### Statistical analysis

Frequencies of pre- and post-discharge complications were analyzed using descriptive statistics. Chi-square tests were used to assess differences between individual complication rates in patients with and without neuromuscular disease. All other analyses were performed in the neuromuscular disease cohort only, comparing those with co-occurring complications and those without. Complications were then cross-tabulated and ranked to identify the most frequently co-occurring complications.

Logistic regression analyses were performed using the Sci-Kit Learn machine learning package in Python (version 1.1.2, Python Software Foundation, https://www.python.org/) to identify patient and treatment factors associated with the risk of concurrent complications and the risk of postoperative infection. Patients with neuromuscular scoliosis with one or no complications were used as the comparison group in the analysis. Variables with a *p*-value < 0.10 on univariate analysis and additional variables of clinical importance were used in the multivariate analysis.

Pearson and point-biserial correlation analyses were used to identify relationships between the number of perioperative complications and the secondary outcomes of hospital stay, new patient ventilation, days on mechanical ventilation, readmissions, non-home discharge, and death.

## Results

### Cohort overview

**Fig. 1:**
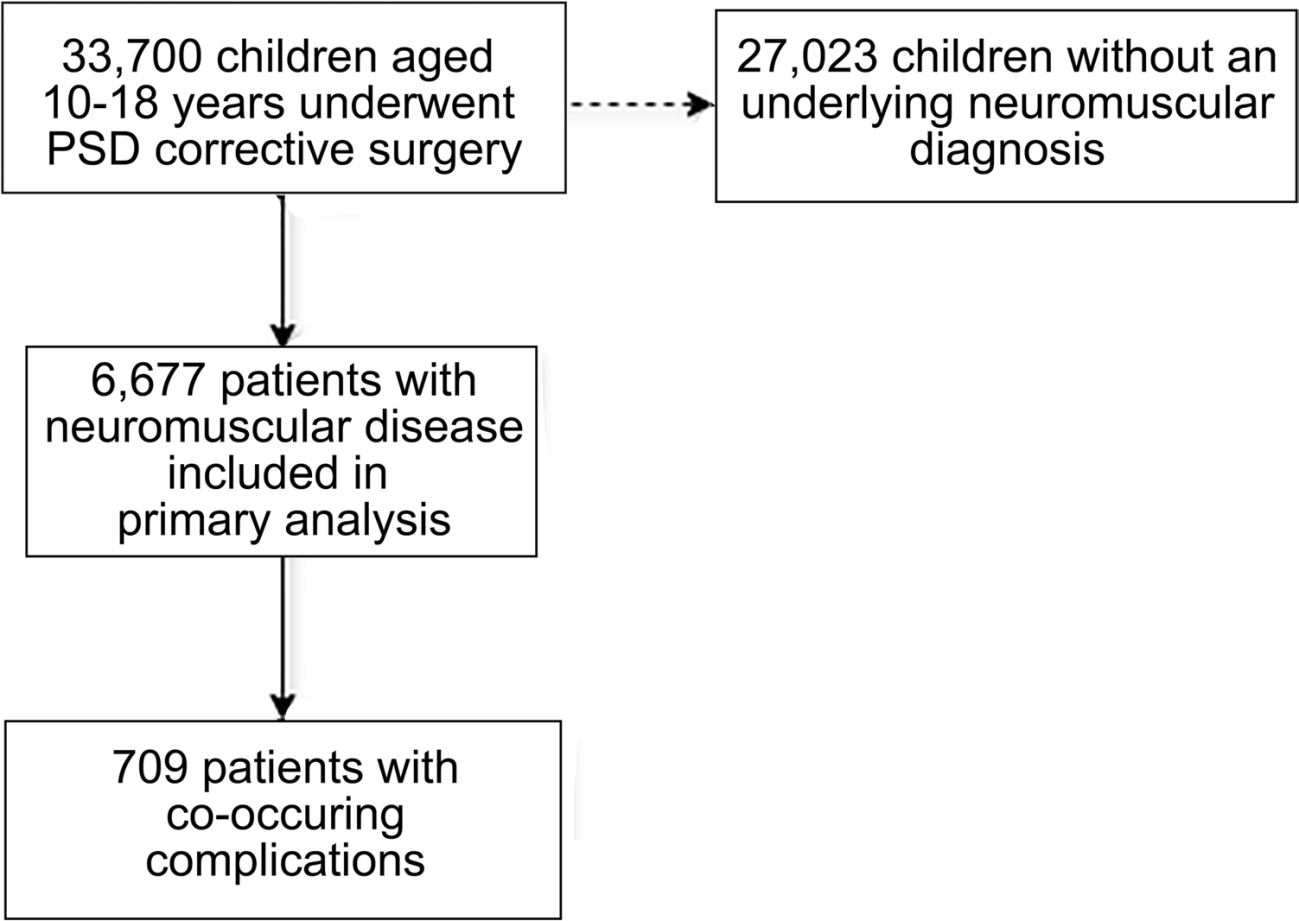
Flow diagram demonstrating the cohort of children included in the study

**Fig. 2:**
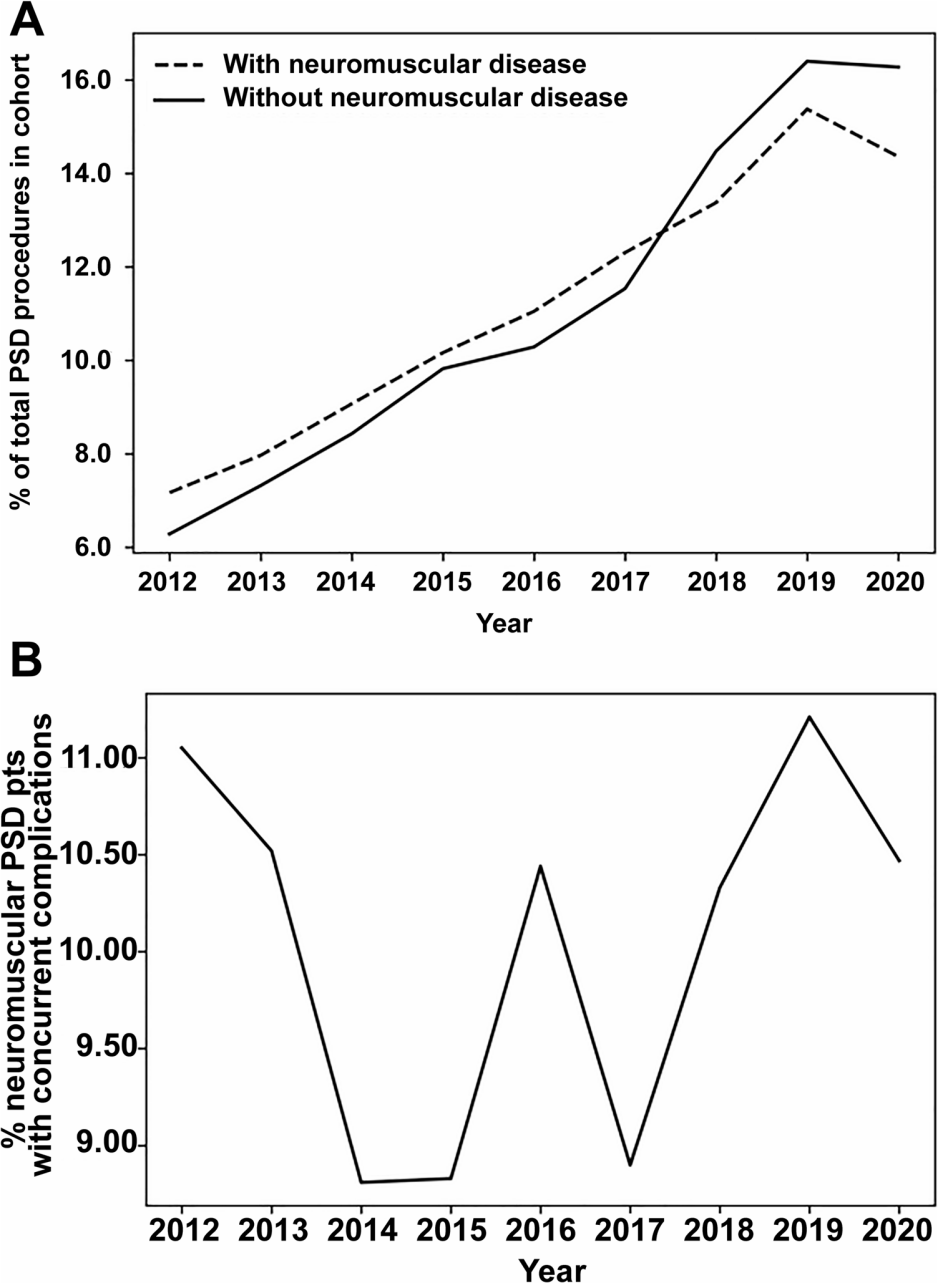
**A** Percentage of pediatric spinal deformity operations performed over the study period for children with neuromuscular disease (dotted line) and without neuromuscular disease (solid line). **B** Percentage of concurrent complications over the study period for children with neuromuscular disease

A total of 6677 children with neuromuscular scoliosis and 27,023 children without neuromuscular diagnoses who underwent spinal fusion surgery were identified (Fig. 1). The median age of the neuromuscular cohort was 13.8 years [interquartile range (IQR) 12.3–15.4], 51.4% had a body mass index < 18.5 kg/m^2^, and 53.7% were female. Among patients with neuromuscular disease, 6.1% underwent < 6-level posterior fusion, 23.4% underwent 7- to 12-level posterior fusion, 67.8% underwent ≥ 13-level posterior fusion, and 2.8% underwent anterior fusion (Table 1). Secondary pelvic fixation was performed in 2105 (31.5%) children in conjunction with their initial spinal fusion surgery. The number of procedures performed and co-occurring complications for spinal correction from 2012 through 2020 can be seen in Fig. 2a, b, respectively.

**Table 1:** Baseline demographic, clinical, and surgical variables for children with neuromuscular disease undergoing spinal deformity correction (*n* = 6677)

Variable	Value^a^
Median age (years)	13.8 (IQR: 12.3–15.4)
Female	3585 (53.7%)
Race	
White	4619 (69.2%)
Black	911 (13.6%)
Asian	218 (3.3%)
American Indian or Alaskan Native	34 (0.5%)
Native Hawaiian/Pacific Islander	14 (0.2%)
Other	8 (0.1%)
Unknown	873 (13.1%)
BMI	
Underweight	3429 (51.4%)
Normal	2358 (35.3%)
Overweight	828 (12.4%)
Obese	62 (0.9%)
Impaired cognitive status at time of surgery	4264 (63.9%)
Nutritional support at time of surgery	1932 (28.9%)
Oxygen support at time of operation	367 (5.5%)
Gastrointestinal disease	1799 (26.9%)
Ventilator dependence	603 (9.0%)
History of asthma	971 (14.5%)
History of bronchopulmonary disorder	982 (14.7%)
Cardiac risk factors	
None	5610 (84.0%)
Minor	513 (7.7%)
Major	509 (7.6%)
Severe	45 (0.7%)
Seizure disorder	2399 (35.9%)
Cerebral palsy	2575 (38.6%)
Structural CNS abnormality	2192 (32.8%)
Hematologic disorder	323 (4.8%)
Inotropic support at time of operation	81 (1.2%)
ASA status^b^	
I	251 (3.8%)
II	1306 (19.6%)
≥ III	5109 (76.5%)
Procedure	
Arthrodesis, posterior, ≤ 6 vertebral segments	407 (6.1%)
Arthrodesis, posterior 7–12 segments	1559 (23.4%)
Arthrodesis, posterior ≥ 13 segments	4527 (67.8%)
Arthrodesis, anterior, 2–3 segments	28 (0.4%)
Arthrodesis, anterior, 4–7 segments	107 (1.6%)
Arthrodesis, anterior, ≥ 8 segments	49 (0.7%)
Pelvic fixation^c^	2105 (31.5%)
Median total operation time (min)	317 (IQR: 246–403)

IQR, interquartile range; BMI, body mass index; CNS, central nervous system; ASA, American Society of Anesthesiologists

^a^Values reported as number (%) unless otherwise indicated

^b^Eleven without ASA classification assigned

^c^Secondary procedure code

### Outcomes

The median length of hospital stay for the neuromuscular cohort was 5 days [IQR 4–7 days] (Table 2). After surgery, 1328 (19.9%) children required mechanical ventilation (mean days 2.3 ± 6.5), of which 891 (13.3%) were not on a ventilator at baseline. Overall, 59 patients required > 30 days of ventilator support, 43 of whom were not on a ventilator at baseline.

**Table 2:** Outcomes for children with neuromuscular disease undergoing spinal deformity correction (*n* = 6677)

Outcome	Value^a^
Perioperative complications	5467 (81.9%)
Concurrent complications	709 (10.6%)
Median length of hospital stay (days)	5 (IQR: 4–7)
Hospitalization > 30 days	97 (1.5%)
Postoperative ventilation support	1328 (19.9%)
Postoperative ventilation support (not ventilated at baseline)	891 (13.3%)
Mean days on mechanical ventilation (± SD)	2.3 ± 6.5
> 30 days on mechanical ventilation	59 (0.9%)
Non-home discharge	413 (6.2%)
Rehabilitation	194 (2.9%)
Skilled nursing facility	113 (1.7%)
Acute care	54 (0.8%)
Unknown	52 (0.8%)
Home discharge	6246 (93.6%)
Readmission within 30 days	570 (8.5%)
Death within 30 days	25 (0.4%)

SD, standard deviation

^a^Values reported as number (%) unless otherwise indicated

At discharge, 6246 (93.6%) patients were discharged home, 194 (2.9%) to rehabilitation, 113 (1.7%) to a skilled nursing facility, 54 (0.8%) to separate acute care, and 52 (0.8%) were unreported. Fourteen patients who required postoperative ventilatory support were discharged to an acute care facility. Twenty-five (0.4%) patients died within 30 days of surgery, including 15 with a history of seizures; 15 of the deceased patients were on a ventilator postoperatively and 10 were not.

### Complication overview

The overall perioperative complication rate in the neuromuscular scoliosis group was 81.9%, and the overall concurrent complication rate (two or more complications) was 10.6% (71.3% of children experienced one complication, 7.0% experienced two complications, 2.5% experienced three complications, and 1.2% experienced four or more complications) (Table 3). Table 4 demonstrates the frequency of specific complications in the neuromuscular and non-neuromuscular cohorts. In patients with neuromuscular scoliosis, the body systems most affected were hematologic (> 80%) and pulmonary (5%). The most encountered complication was bleeding/transfusion (80.0%); the median volume of blood transfused was 340 mL for the entire neuromuscular cohort. Among all complications, only cerebrovascular accident/stroke or intracranial hemorrhage and nerve injury were not significantly more common in the neuromuscular scoliosis cohort.

**Table 3:** Number of patients in the neuromuscular cohort (*n* = 6677) experiencing complications

Number of complications	*n* (%)
0	1210 (18.1%)
1	4758 (71.3%)
2	465 (7.0%)
3	166 (2.5%)
4	54 (0.8%)
5	19 (0.3%)
6	5 (0.1%)

**Table 4:** Perioperative complication rates of patients with (*n* = 6677) and without (*n* = 27,023) a neuromuscular disorder

Perioperative complication	Neuromuscular disorder (*n* = 6677)	Non-neuromuscular disorder (*n* = 27,023)	*p* value^a^
Bleeding/transfusions	5342 (80.0%)	18,070 (66.9%)	< 0.001
Urinary tract infection	138 (2.1%)	94 (0.4%)	< 0.001
Pneumonia	195 (2.9%)	126 (0.5%)	< 0.001
Vein thrombosis requiring therapy	24 (0.4%)	24 (0.1%)	< 0.001
Superficial incisional SSI	115 (1.7%)	157 (5.8%)	< 0.001
Deep incisional SSI	129 (2.1%)	123 (0.5%)	< 0.001
Organ/space SSI	52 (0.8%)	35 (0.1%)	< 0.001
Deep wound disruption/dehiscence	81 (1.2%)	102 (0.4%)	< 0.001
Unplanned intubation	147 (2.2%)	73 (0.3%)	< 0.001
Progressive renal insufficiency	9 (0.1%)	13 (0.05%)	0.023
Acute renal failure	5 (0.1%)	5 (0.02%)	0.045
Coma > 24 h	3 (0.04%)	0 (0%)	0.006
CVA/stroke or intracranial hemorrhage	3 (0.04%)	5 (0.02%)	0.412
Seizure disorder	14 (0.2%)	8 (0.03%)	< 0.001
Nerve injury	14 (0.2%)	95 (0.4%)	0.088
Cardiac arrest requiring CPR	31 (0.5%)	13 (0.05%)	< 0.001
*C. diff* colitis	22 (0.3%)	18 (0.1%)	< 0.001
Sepsis	133 (2.0%)	71 (0.3%)	< 0.001
Septic shock	30 (0.5%)	15 (0.1%)	< 0.001
Central line-associated bloodstream infection	5 (0.1%)	2 (0.01%)	0.003
Death	25 (0.4%)	8 (0.03%)	< 0.001

SSI, surgical site infection; CVA, cerebrovascular accident; CPR, cardiopulmonary respiration; *C. diff*, Clostridium difficile

^a^Compared using chi-square analysis of independence

The overall complication rate was higher in children who underwent pelvic fixation (91.4% vs. 77.5%, *p* < 0.001), with a higher median blood loss (511 mL vs. 340 mL, *p* < 0.001) and thus rates of transfusion (89.9% vs. 77.4%, *p* < 0.001) and longer median operative time (352.5 vs. 300.5 min, *p* < 0.001).

### Complication co-occurrences

Seven hundred and nine (10.6%) patients with neuromuscular scoliosis experienced more than one complication. When assessing new postoperative complications by body system, 42.0% of concurrent complications were hematologic/pulmonary, 45.1% hematologic/wound-related, and 33.3% hematologic/infectious. Table 5 demonstrates the most commonly co-occurring complications, which were led by transfusion/pneumonia (23.8%) and transfusion/unplanned reintubation (18.2%). With transfusion excluded, pneumonia/unplanned reintubation (7.8%) and deep wound surgical site infection/septic shock (6.5%) were the most common co-occurring combinations (Table 5).

**Table 5:** Top 10 most frequent co-occurring perioperative complications (*n* = 709) in the neuromuscular PSD patient cohort (*n* = 6677)

Top 10 perioperative co-occurring complications	Rate
Bleeding/transfusions and pneumonia	169 (23.8%)
Bleeding/transfusions and unplanned reintubation	129 (18.2%)
Bleeding/transfusions and septic shock	119 (16.8%)
Bleeding/transfusions and urinary tract infection	117 (16.5%)
Bleeding/transfusions and deep wound SSI	116 (16.4%)
Bleeding/transfusions and superficial incisional SSI	93 (13.1%)
Bleeding/transfusions and deep wound disturbance/dehiscence	66 (9.3%)
Pneumonia and unplanned reintubation	55 (7.8%)
Deep wound SSI and septic shock	46 (6.5%)
Bleeding/transfusions and organ/space SSI	45 (6.4%)

SSI, surgical site infection

### Factors associated with co-occurring complications

Table 6 presents the univariate and multivariate analysis identifying surgical and clinical risk factors for co-occurring complications. Three surgical treatment factors—fusion ≥ 13 levels (odds ratio (OR) 1.42 [95% confidence interval (CI) 1.13–1.79]), operative time ≥ 400 min (OR 1.49 [1.25–1.75]), and pelvic fixation (OR 1.21 [1.01, 1.44]) independently predicted concurrent complications. The variance inflation factor between these variables is 1.34, implying low collinearity.

**Table 6:** Univariate logistic regression analysis demonstrated unadjusted associations with co-occurring complications

Variable	Univariate analysis		Multivariate analysis	
	OR [95% CI]	*p*-value	OR [95% CI]	*p*-value
Age (continuous)	0.94 [0.91–0.98]	**0.003**	0.99 [0.95–1.04]	0.85
Race				
White	Reference			
Black or African American	1.08 [0.86–1.35]	0.52		
Asian	0.87 [0.55–1.40]	0.57		
American Indian or Alaska Native	1.15 [0.40–3.29]	0.79		
Native Hawaiian or other Pacific Islander	0.66 [0.09–5.09]	0.69		
Unknown/not reported	2.88 [0.58–14.31]	0.2		
Body mass index				
Normal	Reference			
Underweight (< 18.5)	1.13 [0.95–1.34]	0.17	0.87 [0.72–1.04]	0.13
Overweight	1.05 [0.81–1.37]	0.7		
Obese	1.74 [0.88–3.48]	0.11		
Premature birth	1.12 [0.95–1.32]	0.19		
Preoperative ventilator dependence	1.62 [1.28–2.05]	** < 0.001**	1.06 [0.80–1.40]	0.70
ASA classification ≥ III	18.18 [4.51–73.28]	** < 0.001**	1.73 [1.27–2.37]	< 0.001
History of asthma	1.72 [1.41–2.08]	** < 0.001**	1.09 [0.88–1.34]	0.45
History of bronchopulmonary dysplasia/chronic lung disease	1.86 [1.54–2.25]	** < 0.001**	1.18 [0.96–1.46]	0.12
Preoperative oxygen support	2.05 [1.56–2.70]	** < 0.001**	1.21 [0.89–1.66]	0.22
Preoperative tracheostomy	1.37 [1.00–1.88]	0.05	0.62 [0.43–0.89]	**0.009**
Structural pulmonary/airway abnormalities	1.88 [1.57–2.26]	** < 0.001**	1.24 [1.01–1.52]	**0.04**
Gastrointestinal disease	2.03 [1.73–2.39]	** < 0.001**	1.05 [0.87–1.26]	0.63
Previous cardiac surgery	1.24 [0.93–1.67]	0.15		
Impaired cognitive status	3.31 [2.69–4.07]	** < 0.001**	1.80 [1.41–2.30]	** < 0.001**
Minor cardiac risk factors	1.34 [1.10–1.63]	**0.004**	1.15 [0.93–1.42]	0.20
Seizure disorder	2.42 [2.07–2.84]	** < 0.001**	1.36 [1.12–1.67]	**0.002**
Cerebral palsy	1.87 [1.60–2.19]	** < 0.001**	0.87 [0.72–1.07]	0.19
Structural CNS abnormality	1.71 [1.46–2.00]	** < 0.001**	1.18 [0.99–1.40]	0.06
Preoperative ostomy	2.78 [2.31–3.35]	** < 0.001**	1.02 [0.67–1.54]	0.93
Nutritional support	2.74 [2.34–3.21]	** < 0.001**	1.34 [1.08–1.72]	**0.009**
Hematologic disorder	2.01 [1.50–2.69]	** < 0.001**	1.40 [1.03–1.91]	**0.03**
Congenital malformations	1.63 [1.38–1.92]	** < 0.001**	1.20 [1.01–1.44]	**0.04**
Low preoperative albumin	1.37 [0.76–2.45]	0.3		
Procedure (segments)				
Arthrodesis, posterior (≤ 6)	Reference			
Arthrodesis, posterior (7–12)	0.8 [0.49–1.29]	0.36		
Arthrodesis, posterior (≥ 13)	2.51 [1.63–3.85]	** < 0.001**		
Arthrodesis, anterior (2–3)	3.63 [1.26–10.42]	**0.017**		
Arthrodesis, anterior (4–7)	2.94 [1.49–5.78]	**0.002**		
Arthrodesis, anterior (≥ 8)	1.09 [0.31–3.77]	0.89		
Surgical time > 400 min	1.73 [1.47–2.04]	< 0.001	1.49 [1.25–1.75]	< 0.001
Fusion ≥ 13 levels	2.59 [2.11–3.17]	** < 0.001**	1.42 [1.13–1.79]	**0.003**
Pelvic fixation (secondary procedure)	1.98 [1.69–2.32]	** < 0.001**	1.21 [1.01–1.44]	**0.03**

Boldface font indicates statistical significance at *p* < 0.05

CNS, central nervous system; ASA, American Society of Anesthesiologists

Significant patient-specific risk factors were an American Society of Anesthesiologists (ASA) physical status classification ≥ 3 (OR 1.73 [1.27–2.37]), structural pulmonary/airway abnormalities (OR 1.24 [1.01–1.52]), impaired cognitive status (OR 1.80 [1.41–2.30]), seizure disorder (OR 1.36 [1.12–1.67]), hematologic disorder (OR 1.40 [1.03–1.91]), preoperative nutritional support (OR 1.34 [1.08–1.72]), and congenital malformations (OR 1.20 [1.01–1.44]).

Children with existing tracheostomy were less likely to experience concurrent complications (OR 0.62 [0.43–0.89]). Diagnoses included with each of the categories can be found in Online Resource 1.

### Factors associated with infectious complications

Additional multivariate logistic modeling demonstrated that hematologic disorder (OR 1.85 [1.13–3.04]), impaired cognitive status (OR 2.28 [1.4–3.7]), and pre-existing gastrointestinal disease (OR 1.55 [1.10–2.19]) were significantly associated with infection. Operative time ≥ 400 min (OR 1.43 [1.04–1.96]) also predicted infection.

### Co-occurring complications and correlations with clinical outcomes

A greater number of co-occurring complications was correlated with requiring postoperative ventilation support (*r* = 0.12, *p* < 0.001) and longer duration of mechanical ventilation (*r* = 0.24, *p* < 0.001).

Having concurrent complications was also significantly associated with the outcomes of hospital readmission (*r* = 0.36, *p* < 0.001), non-home discharge (*r* = 0.18, *p* < 0.001), and death (*r* = 0.22, *p* < 0.001). The correlation coefficient for total hospital stay and co-occurring complications was − 0.002 (*p* = 0.003).

## Discussion

Pediatric patients with neuromuscular scoliosis experience higher rates of having a single complication than their idiopathic counterparts [24, 27, 29], suggesting that clustered complications are also more frequent. As the frequency of surgery in this population has increased [33], efforts to improve the delivery of care require an understanding of the patients most at risk in this group. In this study, we identified procedural and baseline patient characteristics associated with clustering complications. We found an overall complication rate of 81.9%, which is significantly higher than previously reported, and a concurrent complication rate of 10.6%. We also demonstrated that concurrent complications were associated with non-home discharge, mechanical ventilation after surgery, hospital readmission within 30 days, and death.

A similar study in patients with adult spinal deformity found a co-occurring complication rate of 6.3% [5]. In the adult population, the most commonly occurring concurrent complications were transfusion/urinary tract infection (24.3%) followed by transfusion/pneumonia (17.7%). By comparison, 10% of children with neuromuscular disease experienced co-occurring complications, most commonly transfusion/pneumonia (23.8%) and transfusion/reintubation (18.2%). Although these are very different populations, similarities in sources of morbidity (infectious, pulmonary, hematologic) and surgical risk factors (operative time and fusion length) are cause for comparison. Also using the NSQIP-P data set, Malik et al. [20] studied the frequency and timing of complications in children after posterior spinal fusion and determined particular times at which to be aware of the occurrence of certain complications. The current investigation provides further understanding and awareness of which complications occur in tandem to allow for appropriate anticipation and management.

The well-described pulmonary complications associated with neuromuscular disease are secondary to diminished pulmonary reserve which is often a trait of patients with neuromuscular scoliosis with curves in the operative range. These patients are more likely to develop pulmonary infections [11, 25, 27] and to require subsequent readmission to the hospital after spinal fusion [22]. Martin et al. [22] also found that structural pulmonary abnormalities independently predicted readmission to the hospital after spinal deformity surgery, as also shown in our multivariate analysis. Similarly, Fruergaard et al. [11] studied 1310 patients undergoing primary surgery for pediatric spinal deformity and found pulmonary complications were the primary reason for extended length of stay in the group of patients with neuromuscular disease. They concluded that readmission after pediatric spine surgery in all patient cohorts is highly related to pulmonary causes, including neuromuscular deformity (OR 4.4). As a result, early mobilization, pulmonary physiotherapy, and judicious use of pain medications to prevent aspiration events have been described to avoid adverse pulmonary outcomes in this group [9]. Interestingly, in our study, children who underwent preoperative tracheostomy were less likely to have experienced concurrent complications (OR 0.62 [0.43–0.89]), a reflection of better airway clearance and pulmonary toilet in this subset of children.

Additional characteristics suggesting a heightened risk for postoperative complications include impaired cognitive status and seizure disorder. These factors are not infrequently encountered in patients with neuromuscular disease, who may have both intrinsic risk factors. Although these cannot be modified, preemptive stratification is helpful for informed consent and perioperative preparation [13]. Preoperative malnutrition has also been linked to higher rates of complications, including infections [30]. Although our findings suggest that preoperative nutritional support is associated with a higher occurrence of concurrent complications, it is likely that these results are incidental and related to the weakened physiologic state of these children (e.g., anemic, immunocompromised).

Three surgical factors (fusion ≥ 13 levels, operative time ≥ 400 min, pelvic fixation) independently predicted co-occurring complications. These three variables are related but demonstrated limited collinearity, indicating their individual importance. Pugely et al. [27] also reported that longer operative time (> 4 h) was an independent predictor for complications in children undergoing scoliosis correction surgery, although their report included all children undergoing correction surgery*.* Similarly, Toll et al. [32] demonstrated that longer intraoperative time predicted complications in a cohort of 102 patients with neuromuscular scoliosis. Length of surgery is likely a reflection of the complexity of the deformity and subsequent surgery. As demonstrated in this series, pelvic fixation is associated with greater blood loss and longer operative time because of the need for more extensive soft tissue dissection [28]. This study is the first to report that the use of pelvic fixation independently predicts co-occurring complications in a cohort of children with neuromuscular disease undergoing spinal fusion. Measures to reduce surgical time to modify one of the procedural risk factors include the involvement of two attending surgeons in the procedure, which is common in many centers.

Children with an ASA classification ≥ 3 were 1.80 times more likely to experience concurrent complications. The importance of ASA grading in adult perioperative outcomes is well established, but the body of work in children is less robust. Parallel to our findings, Basques et al. [3] found that ASA ≥ 3 was the only independent risk factor for any adverse event (relative risk 2.2, *p* = 0.012) in a cohort of 940 patients with neuromuscular scoliosis. Children with congenital malformation had 1.23 times higher odds of concurrent complications after spinal fusion surgery. Systemic congenital malformations are associated with spinal anomalies and vice versa; thus, a high index of suspicion is necessary, and any relationship should be investigated before surgery [26].

Inclusion of blood transfusion as a “complication” is controversial because transfusion is expected in most surgeries involving neuromuscular pediatric spinal deformity. NSQIP-P defines blood transfusions as intraoperative or postoperative acute blood loss anemia requiring the administration of blood products. There is a separate variable for preoperative transfusion within 48 h of the procedure, but this was not included as a complication for the purposes of this analysis. Because blood transfusions influence the overall complication profile, we believe it important to account for this. Additionally, in this analysis, the presence of a pre-existing hematologic disorder incurred a 1.44 times higher risk of concurrent complications. Although NSQIP-P does not describe the specific hematologic disorder, common nonmalignant disorders are those related to nutritional anemias (megaloblastic anemia, hemoglobinopathies/thalassemia, or sickle cell disease) or acquired secondary to medication [6]. Preoperative anemia is known to carry a higher odds of surgical site infection [4]. Interestingly, our analysis did not reveal perioperative blood transfusions as an independent predictor of infection, although other studies suggest otherwise. A meta-analysis by Hill et al. [12] observed that, across various procedures, postoperative allogenic blood transfusions were associated with a significantly increased risk of postoperative bacterial infections (common OR 5.26 [5.03–5.43]). Fisahn et al. [10] also reported a similar association between allogenic blood transfusions and infectious complications in adults undergoing major spinal surgery (36% in patients undergoing transfusion vs. 10% otherwise; *p* = 0.03). The specific causative factor of this association remains unclear; however, it is evident that allogenic blood leads to a greater degree of postoperative immunosuppression than autologous transfusions or no transfusion at all. Despite no independent association, 7 of the 10 most frequently co-occurring complications in this study were hematologic/infectious; thus, adjustment of intraoperative maneuvers such as positioning and maintaining normothermia or techniques such as the use of tranexamic acid, electrocautery, topical hemostatic agents, and hypotensive anesthesia that may help reduce the volume of perioperative bleeding should be considered [23].

In NSQIP-P, the blood transfusion variable is defined by the transfusion of blood products or reinfusion of autologous red blood cell or cell-saver products. The total volume is recorded, but, for the years included in this study, we were unable to determine which transfusion events were autologous/cell-saver, allogeneic; the use of cell saver has been shown to reduce the need for allogeneic blood in spinal deformity correction surgery [18] and should be considered in this population.

On the basis of our findings, we recommend focusing optimizing nonmodifiable (ASA classification ≥ 3, structural pulmonary/airway abnormalities, impaired cognitive status, seizure disorder, hematologic disorder, preoperative nutritional support, and congenital malformations) and surgical risk factors (potentially modifiable) in children with neuromuscular disease requiring spinal fusion surgery. This information can help inform patients/families of the risks of surgery and the potential for clustered complications.

### Limitations

There are several limitations to this investigation. The NSQIP-P database is retrospective, and although spinal fusion procedures are captured, the database was not built for spine surgery patients. Specifically, there is no detailed information regarding the severity of deformity, procedural approaches, or surgical techniques. The procedural codes used here are a modest proxy for risk and severity, although indirect. The use of pelvic fixation is associated with complications, but the code for pelvic fixation—22848—is not included in the ACS NSQIP-P as part of the recorded CPT codes or as part of the spinal deformity participant user file (PUF) [22800, 22802, 22804, 22808, 22810, 22812]. By searching for code 22848 as a secondary procedure code, it was present in 2105 (31.53%) patients with neuromuscular disease; the designation as a secondary code is likely less accurate, and thus reflects an under-representation of this procedure in this population of children and is a limitation of the study.

Furthermore, the NSQIP-P database is based on data collected up to 30 days postoperatively, so longer-term complications were not captured. As such, these variables, such as instrumentation failure (screw breakage, rod fracture)—which is an important postoperative complication, were not considered. However, NSQIP-P accurately captures patient characteristics, treatment factors, and early postoperative complications because it involves comprehensive data review performed by professionally trained surgical clinical reviewers. The American College of Surgeon’s strict data integrity requirements for participating hospitals ensure the NSQIP-P sustains quality data for retrospective reviews such as this one.

## Conclusions

In this study of complication clustering by body system and type in children with neuromuscular disease undergoing spinal fusion surgery, the concurrent complication rate was 10.6%. Surgical time ≥ 400 min, fusion ≥ 13 levels, and pelvic fixation were independently associated with the outcome of co-occurring complications. Having more perioperative complications was associated with non-home discharge, longer mechanical ventilation, readmission, and death. This information can help inform presurgical counseling and multidisciplinary planning by focusing on high-risk children to improve patient selection and surgical outcomes.

### Supplementary Information

Below is the link to the electronic supplementary material.

**Online Resource 1**. Table presenting NSQIP-P reporting criteria for patient comorbidity variables used in co-occurring complications analysis. (DOCX 27 kb)

## Data Availability

Data for this study are available from the NSQIP-P database.

## Abbreviations

NSQIP-P: National Surgical Quality Improvement Program Pediatric
IQR: Interquartile range
OR: Odds ratio

## Declarations

### Author disclaimer

“The views expressed in this article are those of the author and do not necessarily reflect the official policy or position of the Department of the Navy, Department of Defense, nor the U.S. Government.” American College of Surgeons National Surgical Quality Improvement Program and the hospitals participating in the ACS NSQIP are the sources of the data used herein; these groups have not verified and are not responsible for the statistical validity of the data analysis or the conclusions derived by the authors.

### Copyright statement

“I am a military service member. This work was prepared as part of my official duties. Title 17, U.S.C., §105 provides that copyright protection under this title is not available for any work of the U.S. Government. Title 17, U.S.C., §101 defines a U.S. Government work as a work prepared by a military service member or employee of the U.S. Government as part of that person’s official duties.”

## References

[1] Ailon T, Sure DR, Smith JS, Shaffrey CI (2016). Surgical considerations for major deformity correction spine surgery. Best Pract Res Clin Anaesthesiol.

[2] American College of Surgeons (2022) American College of Surgeons National Quality Improvement Program. Participant use data file 2012–2020. https://www.facs.org/quality-programs/data-and-registries/acs-nsqip/participant-use-data-file/. Accessed July 20 2022

[3] Basques BA, Chung SH, Lukasiewicz AM, Webb ML, Samuel AM, Bohl DD, Smith BG, Grauer JN (2015). Predicting short-term morbidity in patients undergoing posterior spinal fusion for neuromuscular scoliosis. Spine (Phila Pa 1976).

[4] Birhanu A, Amare HHMGM, Girma T, Tadesse M, Assefa DG (2022). Magnitude of surgical site infection and determinant factors among postoperative patients, a cross sectional study. Ann Med Surg.

[5] Bortz C, Pierce KE, Brown A, Alas H, Passfall L, Krol O, Kummer NA, Wang E, O'Connell B, Wang C, Vasquez-Montes D, Diebo BG, Neuman BJ, Gerling MC, Passias PG (2021). Frequency and implications of concurrent complications following adult spinal deformity corrective surgery. Spine (Phila Pa 1976).

[6] Chambers HG, Weinstein CH, Mubarak SJ, Wenger DR, Silva PD (1999). The effect of valproic acid on blood loss in patients with cerebral palsy. J Pediatr Orthop.

[7] Coe JD, Arlet V, Donaldson W, Berven S, Hanson DS, Mudiyam R, Perra JH, Shaffrey CI (2006). Complications in spinal fusion for adolescent idiopathic scoliosis in the new millennium. A report of the Scoliosis Research Society Morbidity and Mortality Committee. Spine (Phila Pa 1976).

[8] Farshad M, Weber S, Spirig JM, Betz M, Haupt S (2022). Pelvic fixation in surgical correction of neuromuscular scoliosis. N Am Spine Soc J.

[9] Finkel RS, Mercuri E, Meyer OH, Simonds AK, Schroth MK, Graham RJ, Kirschner J, Iannaccone ST, Crawford TO, Woods S, Muntoni F, Wirth B, Montes J, Main M, Mazzone ES, Vitale M, Snyder B, Quijano-Roy S, Bertini E, Davis RH, Qian Y, Sejersen T, Group SMAC (2018). Diagnosis and management of spinal muscular atrophy: Part 2: pulmonary and acute care; medications, supplements and immunizations; other organ systems; and ethics. Neuromuscul Disord.

[10] Fisahn C, Jeyamohan S, Norvell DC, Tubbs RS, Moisi M, Chapman JR, Page J, Oskouian RJ (2017). Association between allogeneic blood transfusion and postoperative infection in major spine surgery. Clin Spine Surg.

[11] Fruergaard S, Ohrt-Nissen S, Pitter FT, Hoy K, Lindberg-Larsen M, Eiskjaer S, Dahl B, Gehrchen M (2021). Length of stay, readmission, and mortality after primary surgery for pediatric spinal deformities: a 10-year nationwide cohort study. Spine J.

[12] Hill GE, Frawley WH, Griffith KE, Forestner JE, Minei JP (2003). Allogeneic blood transfusion increases the risk of postoperative bacterial infection: a meta-analysis. J Trauma.

[13] Jain A, Sponseller PD, Shah SA, Samdani A, Cahill PJ, Yaszay B, Njoku DB, Abel MF, Newton PO, Marks MC, Narayanan UG, Harms Study G (2016). Subclassification of GMFCS level-5 cerebral palsy as a predictor of complications and health-related quality of life after spinal arthrodesis. J Bone Joint Surg Am.

[14] Jain A, Sullivan BT, Kuwabara A, Kebaish KM, Sponseller PD (2017). Sacral-alar-iliac fixation in children with neuromuscular scoliosis: minimum 5-year follow-up. World Neurosurg.

[15] Kuo BJ, Vissoci JR, Egger JR, Smith ER, Grant GA, Haglund MM, Rice HE (2017). Perioperative outcomes for pediatric neurosurgical procedures: analysis of the National Surgical Quality Improvement Program-Pediatrics. J Neurosurg Pediatr.

[16] Lee CS, Merchant S, Chidambaran V (2020). Postoperative pain management in pediatric spinal fusion surgery for idiopathic scoliosis. Paediatr Drugs.

[17] Lee NJ, Kothari P, Kim JS, Shin JI, Phan K, Di Capua J, Somani S, Leven DM, Guzman JZ, Cho SK (2017). Early complications and outcomes in adult spinal deformity surgery: an NSQIP study based on 5803 patients. Global Spine J.

[18] Liang J, Shen J, Chua S, Fan Y, Zhai J, Feng B, Cai S, Li Z, Xue X (2015). Does intraoperative cell salvage system effectively decrease the need for allogeneic transfusions in scoliotic patients undergoing posterior spinal fusion? A prospective randomized study. Eur Spine J.

[19] Lykissas MG, Crawford AH, Jain VV (2013). Complications of surgical treatment of pediatric spinal deformities. Orthop Clin North Am.

[20] Malik AT, Kim J, Yu E, Khan SN (2019). Timing of complications after posterior spinal fusions in pediatric spine deformity. Spine Deform.

[21] Mannion AF, Elfering A (2006). Predictors of surgical outcome and their assessment. Eur Spine J.

[22] Martin CT, Pugely AJ, Gao Y, Weinstein SL (2015). Causes and risk factors for 30-day unplanned readmissions after pediatric spinal deformity surgery. Spine (Phila Pa 1976).

[23] Mikhail C, Pennington Z, Arnold PM, Brodke DS, Chapman JR, Chutkan N, Daubs MD, DeVine JG, Fehlings MG, Gelb DE, Ghobrial GM, Harrop JS, Hoelscher C, Jiang F, Knightly JJ, Kwon BK, Mroz TE, Nassr A, Riew KD, Sekhon LH, Smith JS, Traynelis VC, Wang JC, Weber MH, Wilson JR, Witiw CD, Sciubba DM, Cho SK (2020). Minimizing blood loss in spine surgery. Global Spine J.

[24] Mohamad F, Parent S, Pawelek J, Marks M, Bastrom T, Faro F, Newton P (2007). Perioperative complications after surgical correction in neuromuscular scoliosis. J Pediatr Orthop.

[25] Murphy NA, Firth S, Jorgensen T, Young PC (2006). Spinal surgery in children with idiopathic and neuromuscular scoliosis. What's the difference?. J Pediatr Orthop.

[26] Passias PG, Poorman GW, Jalai CM, Diebo BG, Vira S, Horn SR, Baker JF, Shenoy K, Hasan S, Buza J, Bronson W, Paul JC, Kaye I, Foster NA, Cassilly RT, Oren JH, Moskovich R, Line B, Oh C, Bess S, LaFage V, Errico TJ (2019). Incidence of congenital spinal abnormalities among pediatric patients and their association with scoliosis and systemic anomalies. J Pediatr Orthop.

[27] Pugely AJ, Martin CT, Gao Y, Ilgenfritz R, Weinstein SL (2014). The incidence and risk factors for short-term morbidity and mortality in pediatric deformity spinal surgery: an analysis of the NSQIP pediatric database. Spine (Phila Pa 1976).

[28] Ramo BA, Roberts DW, Tuason D, McClung A, Paraison LE, Moore HGT, Sucato DJ (2014). Surgical site infections after posterior spinal fusion for neuromuscular scoliosis: a thirty-year experience at a single institution. J Bone Joint Surg Am.

[29] Reames D, Smith J, Fu K, Polly DJ, Ames C, Berven S, Perra J, Glassman S, McCarthy R, Knapp RJ, Heary R, Shaffrey C, Committee SRSMaM (2011). Complications in the surgical treatment of 19,630 cases of pediatric scoliosis: a review of the Scoliosis Research Society morbidity and mortality database. Spine (Phila Pa 1976).

[30] Sandrucci S, Cotogni P, De Zolt PB (2020). Impact of artificial nutrition on postoperative complications. Healthcare (Basel).

[31] Stiel N, Ozden J, Ridderbusch K, Moritz M, Kunkel P, Gulati A, Hagemann C, Mladenov K, Stuecker R, Spiro AS (2020). Pedicle screw instrumentation with or without pelvic fixation in neuromuscular scoliosis: outcome and complications in a series of 37 patients with a minimum 2-year follow-up. Surgeon.

[32] Toll BJ, Samdani AF, Janjua MB, Gandhi S, Pahys JM, Hwang SW (2018). Perioperative complications and risk factors in neuromuscular scoliosis surgery. J Neurosurg Pediatr.

[33] von Heideken J, Iversen MD, Gerdhem P (2018). Rapidly increasing incidence in scoliosis surgery over 14 years in a nationwide sample. Eur Spine J.

