# Supplementary material for: Frequency and predictors of complication clustering within 30 days of spinal fusion surgery: a study of children with neuromuscular scoliosis
Source: Spine Deform. 2024 Feb 9;12(3):727–38. doi: 10.1007/s43390-023-00813-8 (PMC11068681; doi:10.1007/s43390-023-00813-8)
Supplement: Supplementary file 1 — Online Resource 1. Table presenting NSQIP-P reporting criteria for patient comorbidity variables used in co-occurring complications analysis. (DOCX 27 kb) [file 43390_2023_813_MOESM1_ESM.docx]

**Frequency and Predictors of Complication Clustering Within 30 Days of Spinal Fusion Surgery: A Study of Children with Neuromuscular Scoliosis**

**Online Resource 1: NSQIP-P reporting criteria for patient comorbidity variables used in co-occurring complications analysis**

| **Comorbidity Features** | **NSQIP-P Criteria** |
| --- | --- |
| History of asthma | A patient who has a history of chronic reactive airway disease **resulting in**:   - Functional disability in daily activities - Chronic medication requirement - Hospitalization   **Exclusion criteria:**   - Exercise-induced asthma, with an inhaler as needed - Patient uses albuterol daily without a diagnosis of asthma |
| Bronchopulmonary dysplasia/chronic lung disease | Documented diagnosis of bronchopulmonary dysplasia or chronic lung disease, **including**:   - Bronchiectasis - Constrictive obliterative bronchiolitis - Cystic fibrosis with pulmonary involvement |
| Oxygen support at time of operation | Patients receiving additional oxygen by any modality within 24 hours of the procedure.  **Exclusion criteria:**   - Receiving oxygen while only in the operating room or during preoperative holding/transport - Using continuous or bilevel positive airway pressure without supplemental oxygen - On ventilatory support receiving room air |
| Tracheostomy | Presence of a tracheostomy at the time of primary procedure. |
| Structural pulmonary/airway abnormalities | Patient must meet one of the following scenarios:   1. **Current diagnosis at the time of surgery:**  - Neck tumors (e.g., teratoma, cystic hygroma) - Laryngeal cleft - Cricoid/subglottic stenosis - Papillomas/intraluminal tumors - Pierre-Robin/hypoplastic mandible - Obstructive sleep apnea - Mediastinal tumor (e.g., lymphangioma, anterior mediastinal mass) - Tracheal/bronchial stenosis - Bronchogenic/foregut duplication cyst - Congenital diaphragmatic hernia - Diaphragmatic paralysis - Intrathoracic lesion - Pneumatocele - Blebs/bullae - Intrathoracic pulmonary sequestration  1. **Diagnosis was present within 7 days of surgery:**  - Pleural effusion (i.e., empyema, hemothorax, chylothorax) - Pneumothorax  1. **Diagnosis at any time prior to surgery:**  - Laryngomalacia - Vocal cord paralysis - Tracheomalacia - Bronchomalacia - Pulmonary hypoplasia - Pneumonectomy - Lobectomy (2 or more)   **Exclusion criteria:**   - Lobectomy of only one lobe of the lung - Cleft palate/lip - Central apnea - Asthma |
| Developmental delay/impaired cognitive status | Medical record documentation stating the patient is currently not appropriate for developmental age. Delays may result from any etiology, including congenital malformations, acquired structural lesions, traumatic injury, birth asphyxia, metabolic disease, or unknown causes.  **Examples:**   - Down syndrome - Edward syndrome - Patau syndrome - Blindness - Deafness |
| Seizure disorder | Chronic seizure disorder requiring medical and/or dietary management at the time of the primary procedure with or without control.  **Exclusion criteria:**   - Febrile seizures - Seizures due to presence of tumor - Seizures not actively managed for >1 year |
| Cerebral palsy | Documented definitive diagnosis of cerebral palsy at any time. |
| Structural CNS abnormality | Any current or history of CNS abnormality due to structural pathology, CNS infection, or immune-mediated inflammation noted on visual or radiologic exam, **including:**   - Myelomeningocele - Microcephaly - Macrocephaly - Hydrocephalus - Hypotelorism - Trigonocephaly - Dandy-Walker malformation - Arnold-Chiari malformation - Syrinx - Tethered cord - Gray/white matter changes - Absent corpus callosum - Fused ventricles - Aqueductal stenosis - Neural tube defects - Cystic or degenerative lesions of the CNS   **Exclusion criteria:**   - Scoliosis - Abnormality due to tumor or mass |
| Gastrointestinal disease | Patient has a diagnosis of congenital, acquired, or structural intestinal tract disorder involving the esophagus, stomach, small intestine, or colon, **including**:   - Esophageal atresia - Intestinal atresia - Gastroschisis - Omphalocele - Necrotizing enterocolitis - Intestinal stenosis - Tracheoesophageal fistula - Hirschsprung disease - Imperforate anus - Malrotation - Volvulus - Cystic fibrosis with gastrointestinal involvement - Inflammatory bowel disease - Unrepaired pyloric stenosis - Gastroesophageal reflux requiring medication. - Pneumoperitoneum   **Exclusion criteria:**   - Diagnosis of constipation requiring daily medication - Patients taking proton pump inhibitors without a diagnosis of GERD - Patient with complaints of abdominal pain only - Documented diagnosis of appendicitis |
| Steroid use | Patient requiring regular administration of oral or parenteral corticosteroid medication within 30 days of the primary procedure.  **Exclusion criteria:**   - Single dose given within 24 hours of the primary procedure - Betamethasone given to the mother prenatally within 30 days of the primary procedure - Topical, inhaled, or rectal corticosteroids |
| Ostomy | Presence of external fistula and/or previously created ostomy present at the time of the primary procedure.  **Examples**:   - Ventriculostomy (external drainage) - Lumbar drain - Tube thoracostomy - Pericardial drain - Esophagostomy - Gastrostomy - Duodenostomy - Jejunostomy - Gastrocutaneous fistula - Ileostomy - Appendicostomy - Cecostomy - Colostomy - Anal fistula - Nephrostomy - Pyelostomy - Ureterostomy - Vesicostomy - Ileovesicostomy - Ureterovesicostomy - Suprapubic catheter |
| Nutritional support | Enteral feedings via orogastric, nasogastric, nasojejunal, or jejunostomy tubes and/or IV total parenteral nutrition at the time of the primary procedure. |
| Hematologic disorder | Patient has a documented disorder affecting the hematologic system, **including**:   - Sickle cell disease - Thalassemia - Hereditary spherocytosis - Thrombocytopenia - Idiopathic thrombocytopenic purpura - Heparin-induced thrombocytopenia - Neutropenia - Henoch-Schonlein disease - Anemia - Basophilia - Dysfibrinogenemia - Eosinophilia - Vitamin K deficiency - Hemophilia - Von Willebrand disease - Antithrombin III deficiency - Congenital protein C or S deficiency - Disseminated intravascular coagulation - Factor II, V, VII, X, XII deficiencies   **Exclusion criteria:**   - Lab values that indicate hematologic disorder, but without a documented diagnosis - Sickle cell trait |
| Inotropic support | IV inotropic pharmacologic support required at the time of surgery, **including**:   - Dopamine - Dobutamine - Epinephrine - Norepinephrine - Vasopressin - Isoproterenol - Ephedrine - Inamrinone - Milrinone   **Exclusion criteria:**   - Inotropic support started intraoperatively |
| Previous CPR | Patients who had received initiation of cardiac compressions or ECMO within 7 days prior to the primary procedure |
| Previous cardiac surgery/cardiac intervention | Patients who have a history of cardiac surgery or intervention documented in their medical record |
| Cardiac risk factors (minor) | Present cardiac disease or history of cardiac disease identified in the following list:   - Aortic valve stenosis - Atrial septal defect (secundum) - Benign neoplasm of the heart - Bicuspid aortic valve - Cardiac pacemaker - Implanted cardioverter or defibrillator - Cardiac dysrhythmias - Anomalous AV excitation - Atrial fibrillation - AV block - Long & short QT syndrome - Sinoatrial node dysfunction - Supraventricular tachycardia - Wolff-Parkinson White syndrome - Essential hypertension - Mitral valve regurgitation - Mitral valve prolapse - Mitral valve stenosis - Repaired patent ductus arteriosus - Patent foramen ovale - Vascular ring - Ventral septal defect |
| Cardiac risk factors (major) | Present cardiac disease or history of cardiac disease identified in the following list:   - Heart wall aneurysm - Anomalies of great veins - Total/partial anomalous pulmonary venous drainage - Coarctation of the aorta - Interrupted aortic arch - Aortic stenosis - Atrial septal defect (ostium primum, sinus venosus) - AV canal defects - Bacterial endocarditis (acute/subacute) - Cardiomyopathy - Ebstein’s anomaly - Double-outlet right ventricle - Endocardial cushion defects - Heart transplant recipient - Unrepaired patent ductus arteriosus - Acute pericarditis - Pulmonic stenosis - Pulmonary hypertension - Pulmonary valve regurgitation - Sinus of Valsalva fistula - Tetralogy of Fallot - Transposition of great vessels - Ventricular fibrillation |
| Cardiac risk factors (severe) | Present cardiac disease or history of cardiac disease identified in the following list:   - Double-inlet right ventricle - Hypoplastic left heart syndrome - Hypoplastic right ventricle - Mitral valve atresia - Pulmonary valve atresia - Pulmonary vascular obstructive disease - Single ventricle - Tricuspid atresia - Truncus arteriosus |
| Preoperative blood transfusion | Patient received a transfusion of whole blood or packed red blood cells within 48 hours of the primary procedure. |
| Childhood malignancy | Patient with or without a documented malignancy meeting the following inclusion criteria:  **No current or prior history of cancer**   - No documented history of cancer - Negative biopsy   **Past history of cancer**   - History of malignancy but no active disease or no active plan for treatment.   **Current cancer or active treatment of cancer**   - Current cancer diagnosis and/or undergoing active treatment |

CNS, central nervous system; GERD, gastroesophageal reflux disease; IV, intravenous; ECMO, extracorporeal membrane oxygenation; AV, atrioventricular

Developed from information presented in “Chapter 4: ACS NSQIP PEDIATRIC Variables & Definitions” *American College of Surgeons (ACS) National Surgical Quality Improvement Program (NSQIP) Pediatrics Operations Manual*. July 1, 2022.
